# Comparative analysis reveals distinct microbial community profiles and assembly mechanisms in sediments of coastal wetland ecosystems

**DOI:** 10.1128/aem.01159-26

**Published:** 2026-09-23

**Authors:** Linqiong Wang, Zhen Chen, Xinle Li, Zhe Zhao

**Affiliations:** 1 College of Oceanography, Hohai University 12462, Nanjing, China; 2 Jiangsu Province Engineering Research Center for Marine Bio-resources Sustainable Utilization, Hohai University 12462, Nanjing, China; Norwegian University of Life Sciences, Ås, Norway

**Keywords:** coastal wetland ecosystems, microbial communities, ecological networks, assembly pattern

## Abstract

Coastal wetland ecosystems (CWEs), including mangroves, saltmarshes, and seagrasses, support vital biogeochemical cycles mediated by microbes, yet their global microbial diversity, distribution, and potential functions remain elusive. Thus, we analyzed 1,381 16S rRNA gene amplicon data sets targeting the V3–V4 and V4 regions to examine microbial diversity and assembly processes across these habitats. Results revealed significant differences in microbial diversity and composition among these ecosystems, with mangroves exhibiting the highest richness and diversity. The habitat-specific indicator taxa were Rhodothermia, Anaerolineaceae, and SBR1031 in mangroves, *Flavobacteriaceae*, *Burkholderiales*, and *Woeseiaceae* in saltmarshes, and *Desulfosarcinaceae*, *Pseudomonadaceae*, *Firmicutes*, and *Bacillales* in seagrasses through linear discriminant analysis effect size (LEfSe) and random forest analyses. Co-occurrence network analysis revealed a robust structure comprising 1,521 nodes and 64,463 edges, dominated by *Gammaproteobacteria*, *Desulfobacteria*, *Bacteroidia*, and *Desulfobulbia*. KEGG-based functional prediction revealed distinct functional profiles across CWEs. Mangrove communities exhibited higher predicted potentials for carbon fixation and complex hydrocarbon metabolism, whereas seagrass communities showed higher potentials for sulfate metabolism. Saltmarsh communities were enriched in predicted functions related to the degradation of certain aromatic compounds. Using iCAMP, drift was most pronounced in seagrass communities (63.10%), whereas its contribution was lower but remained substantial in mangroves and saltmarshes. Deterministic processes also contributed considerably to community assemblies in mangroves (43.04%) and saltmarshes (44.42%). This underscores the dependence of dominant assembly processes on local environmental contexts. Our findings establish a basis for elucidating the structure and function of microbial communities in CWEs, offering insights for future hypothesis-driven research and enhancing predictive capacity amid growing anthropogenic and climatic pressures.

**IMPORTANCE:**

Coastal wetlands, including mangroves, saltmarshes, and seagrasses, protect shorelines and play a critical role in regulating the global climate. Microbial communities maintain the stability and essential functions of these ecosystems. This study provided the first comprehensive global map of microbial communities in the three ecosystems. By analyzing over 1,381 samples, the results revealed that different habitats relied on different diversity, composition, and dominant taxa. Mangroves showed the highest richness and diversity. KEGG-based functional predictions suggested that microbial communities in the CWEs shared core predicted metabolic functions but differed in their predicted functional potentials. The null model revealed that the assembly process of the three habitats is mainly shaped by stochastic ecological drift. However, deterministic selection also played a significant role in structuring saltmarsh and mangrove communities, suggesting that environmental factors commonly influenced the community assembly. Understanding these patterns is crucial for conservation because it allows scientists to better predict how coastal protection and carbon storage face increasing pressure from human activity.

## INTRODUCTION

Coastal wetland ecosystems (CWEs) are recognized as fundamental blue carbon ecosystems, which include mangroves, saltmarshes, and seagrasses (1). Located in intertidal zones at the interface of land and ocean, CWEs are highly vulnerable to climate change and human activity, which threaten their critical functions, including carbon storage, biodiversity conservation, and climate regulation (2). Given their ecological significance, the conservation and restoration of CWEs have been proposed as key components of climate mitigation strategies, with an emphasis on maintaining and enhancing blue carbon sequestration (3). Microbial communities are the most dominant and diverse organisms in CWEs and support essential ecosystem functions and services (4). Understanding the diversity, structure, and ecological roles of microbial communities is therefore essential for improving our knowledge of the processes that sustain coastal wetland ecosystems.

Microbial communities are indispensable for the health and functioning of CWEs, serving as key drivers of critical biogeochemical processes such as organic matter decomposition, nutrient cycling, and contaminant degradation (5). Furthermore, these microbial communities form intricate social networks within sediments, wherein interactions such as resource competition, cooperative cross-feeding, and chemical communication through secreted compounds collectively influence group behavior and environmental conditions (6). For instance, actively growing species may excrete secondary metabolites that modify the local microenvironment, which in turn affects the growth and relative abundance of other organisms (7, 8). These microbial communities are primarily shaped by geographic and environmental factors within CWEs, reflecting their adaptive dynamics in response to varying environmental conditions.

Specifically, mangrove sediments, characterized by predominantly anaerobic conditions, exhibit high and variable levels of redox potential and sulfur sources. Consequently, the abundance of sulfate-reducing bacteria is typically elevated in these environments, driving the crucial conversion of sulfate into sulfide (9–11). For example, the diversity of sulfate-reducing bacteria (e.g., *Desulfuromonadales*), which act as major diazotrophic organisms, increases with the age of mangrove restoration. Saltmarshes, subjected to variable tidal inundations and high salinity (~30‰), are dominated by representative plant species such as *Spartina alterniflora, Scirpus mariqueter*, and *Phragmites australis* (12). These plants shape distinct microbial communities enriched with aerobic and facultatively anaerobic chemoheterotrophs (e.g., *Xanthobacteraceae* and *Kiloniellaceae*) and archaea (e.g., *Nitrosopumilaceae*), all of which play integral roles in biogeochemical cycling (13). Meanwhile, seagrasses provide unique stabilizing habitats that support specialized microbial communities, including endophytes residing within the plants, epiphytes on leaves, and rhizosphere microorganisms (e.g., *Actinobacteria*, *Firmicutes*, and *Chloroflexi*) in underlying sediments (14, 15). Notably, these findings provide detailed insights into the distinct microbial characteristics of individual CWE types. It is also important to clarify that microbial communities across the three CWEs are assembled through fundamentally different ecological processes, which help explain the observed patterns of community structure and dynamics (16). Understanding the mechanisms that govern microbial community assembly and function is central to microbial ecology, yet remains a critical knowledge gap.

The assembly processes of microbial community are governed by two main theoretical frameworks. Neutral theory highlights that community assembly is dominantly governed by stochastic processes, including ecological drift and dispersal. In contrast, niche theory argues that community structure is shaped by deterministic processes arising from species traits and environmental conditions (17–19). The assembly of microbial communities in CWEs is investigated through both the neutral and niche theories. However, the relative importance of these two processes remains a subject of debate and varies significantly with different environmental factors. For example, a previous study demonstrated that seagrasses secrete compounds that create selective pressures, causing deterministic processes to dominate microbial community assembly over stochastic processes (20). Likewise, deterministic processes predominantly govern microbial community assembly in southeastern Chinese mangroves, whereas stochastic processes play a more important role in South American mangrove ecosystems (21, 22). Thus, quantifying the relative importance of stochastic and deterministic processes is crucial for understanding the potential mechanisms of microbial community assembly. However, the ecological processes driving microbial community assembly in CWEs have not been systematically characterized on a cross-continental scale.

Therefore, to comprehensively elucidate the role of microbial communities in CWEs, we undertook a global meta-analysis of coastal sediment bacterial microbiomes. Our study leveraged an extensive data set of 1,381 sediment samples of the CWEs from across Asia, Australia, Europe, North America, and South America. This study explicitly addressed three fundamental questions: (i) What are the global patterns and characteristics of microbial community diversity and composition across mangroves, saltmarshes, and seagrasses? (ii) How do the network stability and predicted ecological functions differ among these habitats? (iii) Which main ecological processes govern microbial community assembly across these three coastal environments? By systematically answering these questions, this work aims to significantly advance our understanding of the ecological underpinnings and functionality of CWEs. The findings will offer invaluable, microbe-centric insights into the responses of blue carbon ecosystems, thereby providing essential understanding for guiding informed decisions in wetland ecological restoration.

## MATERIALS AND METHODS

### Origin of microbiome data

#### The three main coastal wetland ecosystems

Coastal wetland ecosystems are transitional ecosystems between terrestrial and marine environments, which play a crucial role in ecological processes. This study focuses on three primary types: mangroves, saltmarshes, and seagrasses, which are bounded by the highest levels of tidal inundation at the terrestrial edge and recognized as the principal blue carbon ecosystems (1). Mangroves are unique woody plant communities, primarily composed of trees and shrubs. They thrive on the intertidal flats of tropical and subtropical coastlines, tolerating regular inundation by saltwater. Saltmarshes are found at the interface of land and sea, characterized by muddy or sandy substrates that are periodically or irregularly submerged by tidal seawater. These environments are dominated by specialized salt-tolerant plants adapted to brackish or saline conditions. Seagrasses consist of marine flowering plants that grow in the intertidal coastal and subtidal zones. Primarily distributed in tropical to temperate regions, they typically inhabit waters less than 25 meters deep. Mangroves are tidally influenced swampy habitats that are mainly restricted to tropical and subtropical coastal regions. Seagrasses occur across six principal distributional realms: the tropical Indo-Pacific, tropical Atlantic, temperate North Pacific, temperate North Atlantic, the Mediterranean, and the temperate Southern Ocean. Salt marshes are highly productive intertidal wetlands predominantly found in temperate climatic zones (23–25). Detailed sampling dates and corresponding metadata for all data sets are summarized in Table S1 and Fig. S1.

#### Coastal sediment sampling in Jiangsu Province, China

Coastal sediment samples were collected from salt marshes in Yancheng City, Jiangsu Province, China. A total of 34 representative sampling sites were selected within the coastal salt marshes of Yancheng City. At each site, surface sediment (0–20 cm depth) was collected in triplicate within a 10 m × 10 m area, and these triplicates were then composited into a single sample. A total of 146 sediment samples were collected from Yancheng, comprising 136 routine seasonal samples (34 sites across four seasons) and 10 additional field replicates from the first campaign. Sediment samples were collected using a stainless-steel Van Veen grab sampler. Each composite sample was immediately packed into airtight sterile polypropylene bags and stored frozen at −20°C onboard until transport to the laboratory. Upon arrival, a portion of each sediment sample was transferred and stored at −80°C for subsequent DNA extraction.

#### Data collection from other studies

**Fig 1:**
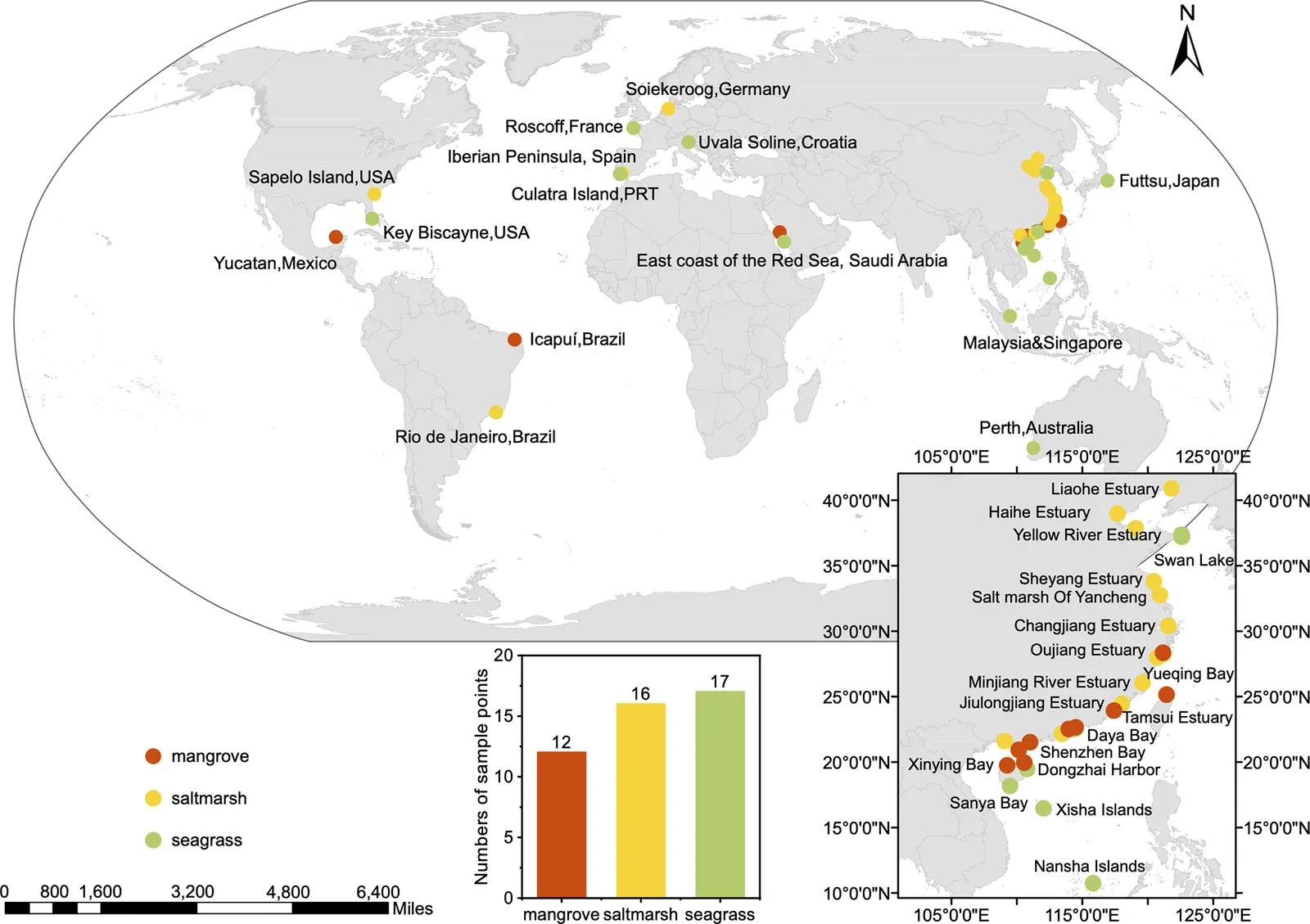
Geographic distribution of the sampling sites across global coastal regions. The map was created in ArcGIS and produced using Copernicus WorldDEM-90 ©DLR e.V. 2010–2014 and ©Airbus Defence and Space GmbH 2014–2018, provided under COPERNICUS by the European Union and ESA; all rights reserved.

We conducted a systematic meta-analysis of publicly available 16S rRNA gene sequencing data on coastal sediment bacterial communities. Adhering to the PRISMA (Preferred Reporting Items for Systematic Reviews and Meta-Analyses) guidelines (26), a thorough literature search for peer-reviewed articles published before December 2024 was performed using Web of Science (Fig. S2). To minimize the confounding effects of depth heterogeneity, only samples explicitly identified as surface sediments (0–20 cm) were included in this study. The search strategy employed the following keyword combinations: “mangrove” OR “seagrass” OR “saltmarsh” OR “coastal sediment” OR “coastal wetland” AND “bacterial community” OR “bacterial diversity” OR “microbial community” OR “biota” OR “bacteria” OR “microbiome.” All retrieved records underwent manual screening to ensure they met the following strict inclusion criteria: (a) Amplicon-based high-throughput sequencing targeting the V3–V4 or V4 hypervariable regions of the 16S rRNA gene. (b) Samples collected specifically from rhizosphere and surrounding coastal sediment. (c) *In situ* field sampling. Raw sequencing data from qualified studies were downloaded from the National Center for Biotechnology Information (NCBI) Sequence Read Archive (SRA) database. This systematic approach resulted in a global data set comprising 1,381 samples from 45 distinct coastal locations (Fig. 1). These locations were distributed across multiple continents, including 32 sites in Asia, one in Oceania, two in Africa, five in Europe, three in North America, and two in South America, which included samples collected from 10 countries. Detailed coordinates and basic information for all collected samples are available in the supplemental material.

#### DNA extraction and 16S rRNA sequencing of the Yancheng coastal saltmarsh sediment samples

Genomic DNA was extracted from 0.25 to 0.5 g of sediment collected from the Yancheng, Jiangsu, China coastal saltmarsh using the FastDNA SPIN Kit for Soil, following the manufacturer’s instructions. The hypervariable V3–V4 regions of the prokaryotic 16S rRNA gene were amplified using the universal primers 341F and 806R. Amplicons were subsequently purified with the QIAquick PCR Purification Kit and subjected to high-throughput sequencing on an Illumina HiSeq 4000 platform (Shanghai Biozeron Biotechnology Co., Ltd).

### Data processing

Raw sequencing data were processed using QIIME 2 (v2024.10). Primer sequences were removed using the q2-cutadapt plugin according to the primer information reported in the original publications. Paired-end reads were merged using FLASH (v1.2.11). Sequence quality profiles were inspected using the q2-demux summarize plugin to determine the trimming and truncation parameters for denoising. For each individual study, amplicon sequence variants (ASVs) were inferred independently using the DADA2 plugin in QIIME 2, including quality filtering, denoising, chimera removal, and feature inference, generating a feature table and representative sequences for each project.

The feature tables and representative sequences from all projects were subsequently merged using the “qiime feature-table merge” and “qiime feature-table merge-seqs” plugins, respectively. ASVs were matched based on 100% sequence identity. The merged feature table was exported as a tab-delimited ASV table, and only ASVs occurring in at least five samples were retained. To reduce systematic technical variation arising from differences between the V3–V4 and V4 hypervariable regions, ComBat-seq batch correction was applied to the merged ASV abundance matrix using the R package “*sva,*” with sequencing region (V3–V4 vs. V4) specified as the batch variable. To evaluate the effectiveness of batch correction, ASVs were aggregated to the family level, and Bray–Curtis-based principal coordinates analysis (PCoA) and permutational multivariate analysis of variance (PERMANOVA) were performed before and after ComBat-seq correction (Fig. S3 and Table S2). These results indicate that the influence of sequencing-region-associated technical variation was substantially reduced, while the major biological patterns among habitats were preserved. Therefore, the merged feature table was exported as a tab-delimited ASV table for downstream analyses.

Taxonomic assignment was performed separately for each project using a naïve Bayesian classifier trained against the SILVA 138 database (https://www.arb-silva.de/), with classifiers constructed to match the corresponding amplified region. For phylogenetic analyses, representative sequences corresponding to the retained ASVs were aligned using MAFFT within the q2-phylogeny pipeline (27). The resulting alignment was refined by masking highly variable and gap-rich positions using the default masking procedure implemented in QIIME 2. A maximum-likelihood phylogenetic tree was then constructed using FastTree 2 (28) and rooted at its midpoint prior to downstream phylogenetic analyses.

### Random forest analysis

To identify key microbial taxa that differentiate the mangrove, saltmarsh, and seagrass ecosystems, a random forest (RF) model was implemented using the ‘randomForest’ package in R. The model was trained using (1,000) classification trees to ensure stable results. The importance of each ASV was evaluated based on the Mean Predictor Importance (MPI), which measures the decrease in classification accuracy when a specific taxon is removed from the model. Taxa with the highest MPI values were identified as the most influential biomarkers for habitat differentiation.

### Network construction

The co-occurrence networks were constructed based on ASV relative abundance data after filtering out ASVs present in fewer than 30% of samples, a step taken to enhance statistical power without substantial ASV loss. Pairwise associations were inferred using Spearman’s rank correlation coefficients, and the significance threshold for network construction was determined automatically using random matrix theory. To evaluate non-random network properties, corresponding random networks preserving the same number of nodes and edges were generated through node rewiring (29). Network topology was characterized using several key metrics, including average degree (avgK, the mean number of connections per node and serves as an indicator of overall network complexity), average clustering coefficient (avgCC, the tendency of nodes to form tightly connected clusters), average geodesic distance (GD, the average shortest path length between node pairs), and harmonic geodesic distance (HD, a modified form of GD that accounts for potential disconnected components within the network). Topological indices for both empirical and random networks were analyzed as previously described (http://ieg4.rccc.ou.edu/mena/).

### Functional annotation

To obtain comprehensive microbial functional profiles from our 16S rRNA gene sequencing data, we performed functional gene prediction using Tax4Fun2. This approach predicts functional potential by linking taxonomic profiles to reference genomes and reconstructing functional annotations based on the KEGG Orthology database (30). Briefly, representative ASV sequences were aligned against the Ref99NR reference database implemented in Tax4Fun2 using BLAST with a minimum sequence identity threshold of 97%. For each sample, functional profiles were inferred by mapping matched reference genomes to KEGG Orthology identifiers and normalizing predicted gene abundances according to the relative abundance of corresponding ASVs. This metric reflects the coverage of functional prediction based on available reference genomes. Samples with assignment rates below 50% were excluded from downstream functional analyses to minimize biases associated with insufficient reference genome representation.

### Microbial community assembly process

A phylogenetic-bin-based null model analysis framework, Infer Community Assembly Mechanisms by Phylogenetic-Bin-Based Null Model Analysis (iCAMP) (31), was employed to quantify the relative importance of ecological processes in shaping the microbial community (32). This framework utilized the β-Net Relatedness Index (βNRI) to infer phylogenetic beta diversity and the Raup-Crick index to infer taxonomic beta diversity. Based on these metrics, the relative importance of five core assembly processes was calculated: heterogeneous selection, homogeneous selection, dispersal limitation, homogenizing dispersal, and ecological drift.

### Statistical analysis

All the statistical analysis were performed based on R version 4.4.1 (http://www.r-project.org). To normalize for uneven sequencing depth, the feature table was first rarefied to a uniform depth. Microbial alpha diversity (Richness, Chao 1, Shannon, and Simpson indexes) was then calculated on this rarefied data using the “phyloseq” package (33). To determine significant differences across different blue carbon ecosystems, a one-way analysis of variance was performed, followed by Tukey’s post-hoc tests for pairwise comparisons. Beta diversity was calculated using the weighted UniFrac distance to characterize phylogenetic and taxonomic variation in community structure. This distance matrix was then visualized using a PCoA plot with the “vegan” package. Statistical significance of differences in community composition was tested using a nested PERMANOVA on the same distance matrix with 999 permutations. Following PERMANOVA, a permutation-based test for homogeneity of multivariate dispersions (PERMDISP) was conducted to ascertain if observed community differences were influenced by variations in dispersion. Linear discriminant analysis effect size (LEfSe) was performed to identify specific microbial taxa that significantly differed in abundance among coastal ecosystem types. Statistical significance was evaluated using the non-parametric Kruskal-Wallis test (α = 0.05). Linear discriminant analysis (LDA) was then applied to estimate the effect size of differentially abundant taxa, and taxa with LDA scores greater than 2.0 were considered significant biomarkers.

## RESULTS

### Diversity and composition of microbial communities are highly differentiated by three coastal wetland ecosystems

**Fig 2:**
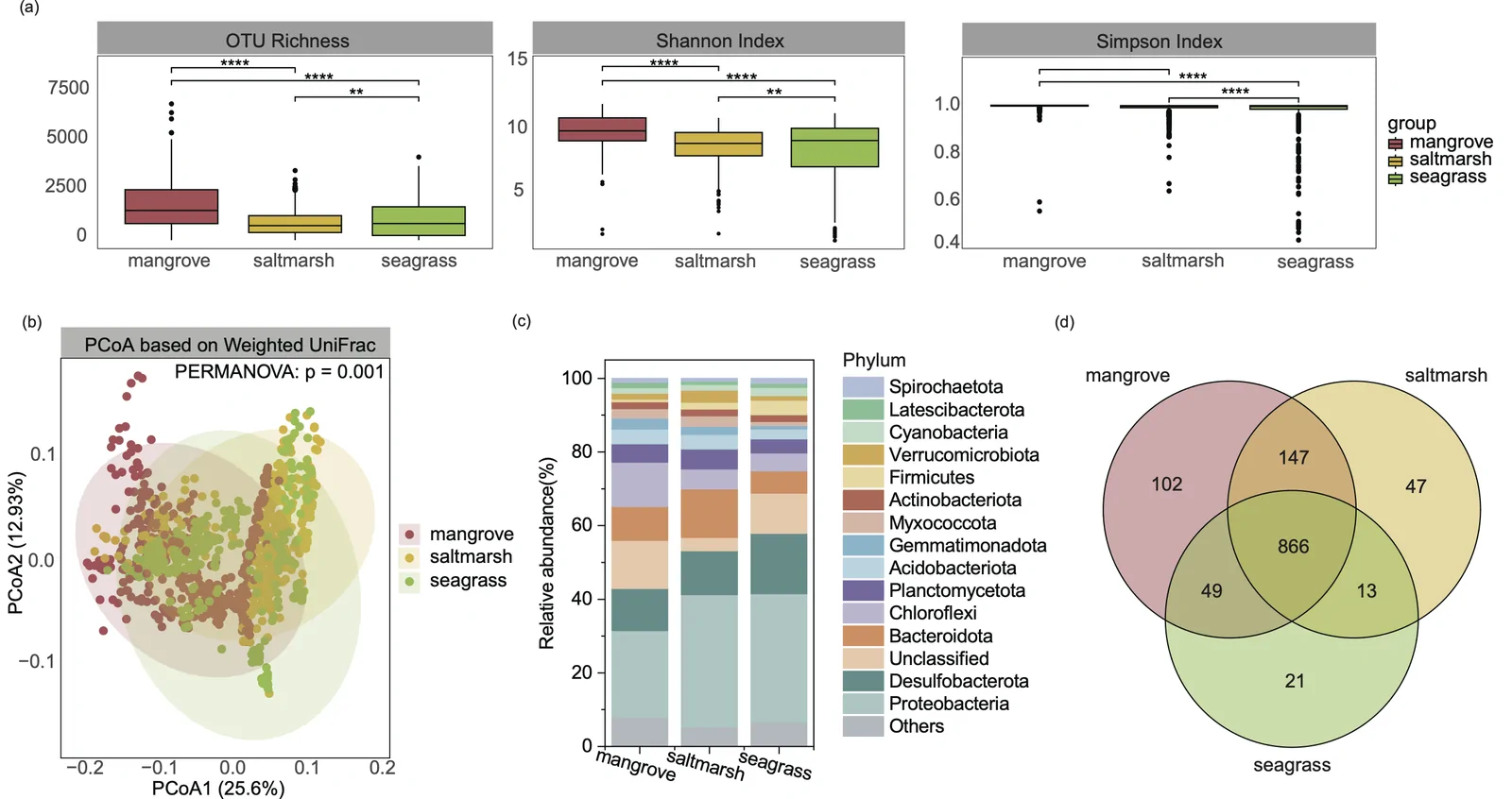
Comparisons of microbial diversity and composition among mangrove, saltmarsh, and seagrass ecosystems. (**a**) Microbial alpha diversity measured by OTU richness, Shannon index, and Simpson index. Significant differences between habitats are indicated by asterisks (determined by pairwise Wilcoxon test). (**b**) Beta diversity visualized via PCoA based on weighted UniFrac distances. PERMANOVA results indicate significant differences in community structure (*P* = 0.001), with ellipses representing 95% confidence intervals. (**c**) Microbial community composition at the phylum level across the three coastal wetlands; only the top 15 phyla are displayed. (**d**) Venn diagram showing the number of shared and unique ASVs among the three habitats. In all panels, colors denote habitat types: red for mangrove, orange/yellow for saltmarsh, and green for seagrass. Statistical significance: *P < 0.05, **P < 0.01, *** *P* < 0.001, ****P < 0.0001.

We analyzed a combined global data set of 1,381 sediment samples from three coastal wetland habitats: mangroves (*n* = 449), saltmarshes (*n* = 475), and seagrasses (*n* = 457). Sequencing of all samples yielded 56,718,154 quality-filtered reads that were clustered into 767,570 ASVs. Significant variations in bacterial alpha diversity were observed across the three habitats (Fig. 2a). Specifically, mangrove sediments exhibited the highest bacterial richness and diversity, with significant differences compared to both saltmarshes (*P* < 0.001) and seagrasses (*P* < 0.001), as confirmed by both Chao1 and Shannon indices (Fig. 2a). Additionally, a significant difference in alpha diversity of bacterial communities was noted between saltmarshes and seagrasses (*P* < 0.05).

A pronounced divergence in bacterial community composition was observed among the three coastal habitats, as indicated by a PERMANOVA (Table S3). PCoA further demonstrated a clear separation, with samples forming distinct clusters according to habitat (Fig. 2b). Notably, bacterial communities in mangrove and seagrass sediments exhibited greater similarity to each other than to those in saltmarshes. At the phylum level, *Proteobacteria* and *Desulfobacterota* were dominant across all habitats (Fig. 2c). *Proteobacteria* accounted for 23.43%, 36.00%, and 34.80% in bacterial communities of mangroves, saltmarshes, and seagrasses, respectively. The abundances of *Proteobacteria* (36.00%) and *Bacteroidota* (13.22%) in saltmarshes were highest among the three habitats (*P* < 0.05).

Apart from *Proteobacteria*, the composition of other major phyla (relative abundance >1%) exhibited clear habitat specificity. In mangrove sediments, key phyla were led by *Chloroflexi* (12.04%), *Desulfobacterota* (11.46%), *Bacteroidota* (9.17%), *Planctomycetota* (5.01%), and *Actinobacteriota* (1.87%). Saltmarsh sediments were characterized by abundant *Bacteroidota* (13.22%), *Desulfobacterota* (11.96%), *Planctomycetes* (5.38%), *Chloroflexi* (5.38%), *Verrucomicrobia* (3.27%), *Actinobacteria* (1.86%), and *Gemmatimonadetes* (2.27%). Seagrasses were dominated by *Desulfobacterota* (16.39%), *Bacteroidota* (6.04%), *Chloroflexi* (4.87%), and *Planctomycetota* (3.84%) (Fig. 2c). Venn diagram analysis further reflected these habitat-driven differences (Fig. 2d). While 866 ASVs were core features shared across all three ecosystems, mangroves harbored the highest number of unique ASVs (102), substantially exceeding those observed in seagrasses and saltmarshes.

### Distinct indicator taxa characterize three coastal wetland ecosystems

**Fig 3:**
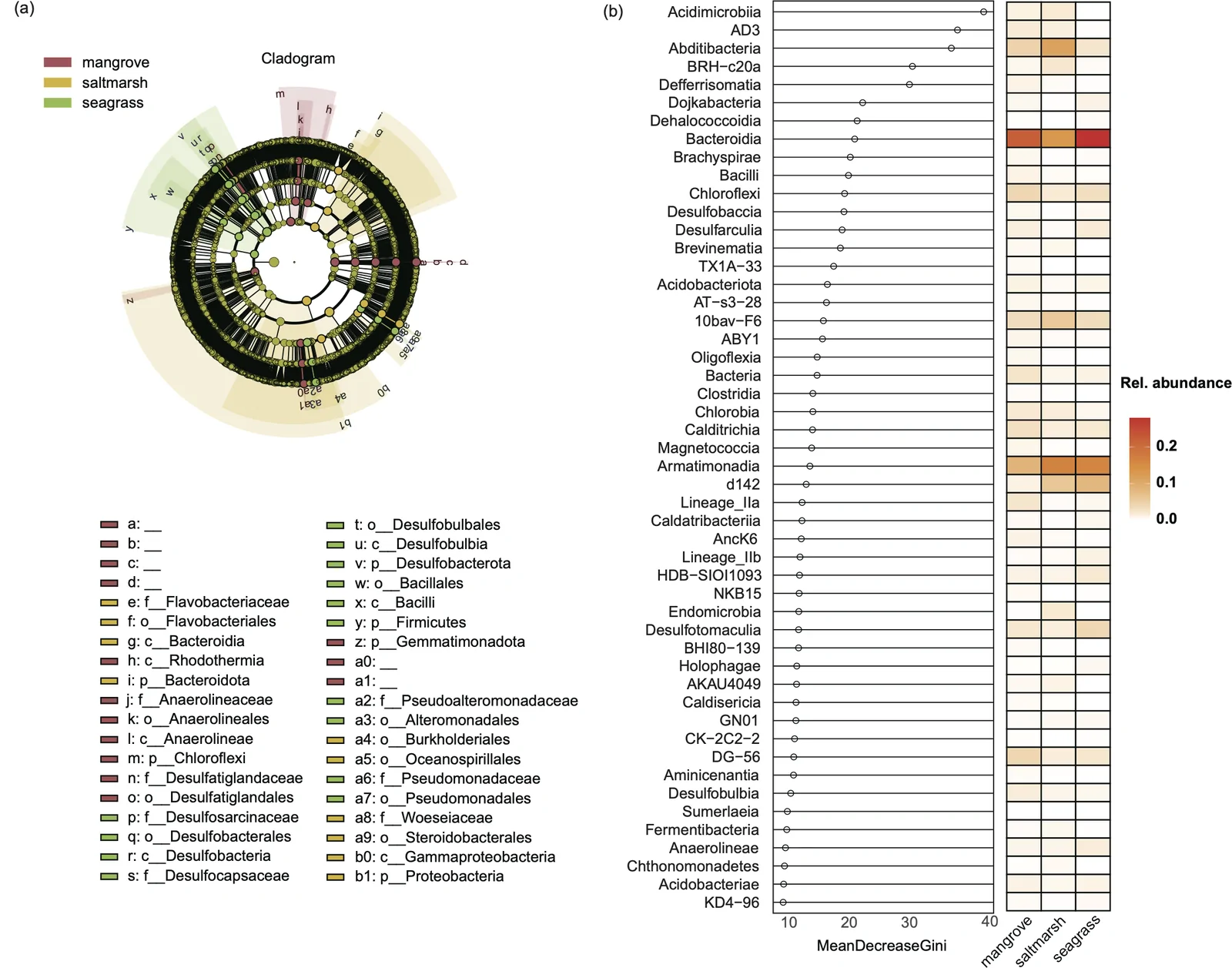
The characteristic microbial taxa in the three coastal habitats. (**a**) LEfSe cladogram identifying phylum- to genus-level indicator taxa among mangroves (red), saltmarshes (orange), and seagrasses (green). Node colors indicate habitat-specific enrichment, and circle sizes are proportional to mean relative abundance. (**b**) Top 50 class-level biomarkers selected by random forest analysis. Bar lengths denote variable importance based on Mean Decrease Gini scores. The accompanying heatmap displays the normalized mean relative abundance of each biomarker across the three coastal habitats (color gradient: light orange to red).

To mitigate the confounding effect of phylogenetic differences, we compared the taxa within the same phylum across different coastal wetlands. Using LEfSe, we identified phylum-level indicator taxa for bacterial communities in mangroves (red branches), saltmarshes (orange branches), and seagrasses (green branches) (Fig. 3a). In mangroves, the indicator taxa at the phylum level were found to be Rhodothermia, Anaerolineaceae, and SBR1031. Conversely, the saltmarsh communities were characterized by the presence of *Flavobacteriaceae*, *Bacteroidia*, *Burkholderiales*, and *Woeseiaceae*. The indicator taxa for seagrasses were distinct, including *Desulfosarcinaceae*, *Pseudomonadaceae*, *Firmicutes*, and *Bacillales*. Moreover, we applied an RF model to identify class-level microbial biomarkers that discriminate among the three CWEs. Based on ten-fold cross-validation error, 67 variables were identified as the optimal set to minimize this error (Fig. S5). As shown in Fig. 3b, biomarkers were identified using the top 50 most abundant classes for mangroves, saltmarshes, and seagrasses. Among these, four classes, *Bacteroidia*, *Armatimonadia*, *10bav-F6*, and *Chloroflexi*, were found to be common biomarkers across all three CWEs. Specifically, habitat-specific biomarkers exhibited distinct enrichment patterns. For saltmarsh ecosystems, the special biomarkers included *Endomicrobia*, *BRH-c20a*, and *Chthonomonadetes*. Mangrove ecosystems were uniquely characterized by the presence of *Defferrisomatia*, *Lineage_lla*, *DG-56*, *AncK6*, and *Oligoflexia*, while seagrasses were distinguished by the three biomarkers, *Desulfotomaculia*, *Lineage_llb*, and *HDB-SIOI1093* (Fig. 3b).

### Enhanced structural complexity and robustness in saltmarsh microbial networks

**Fig 4:**
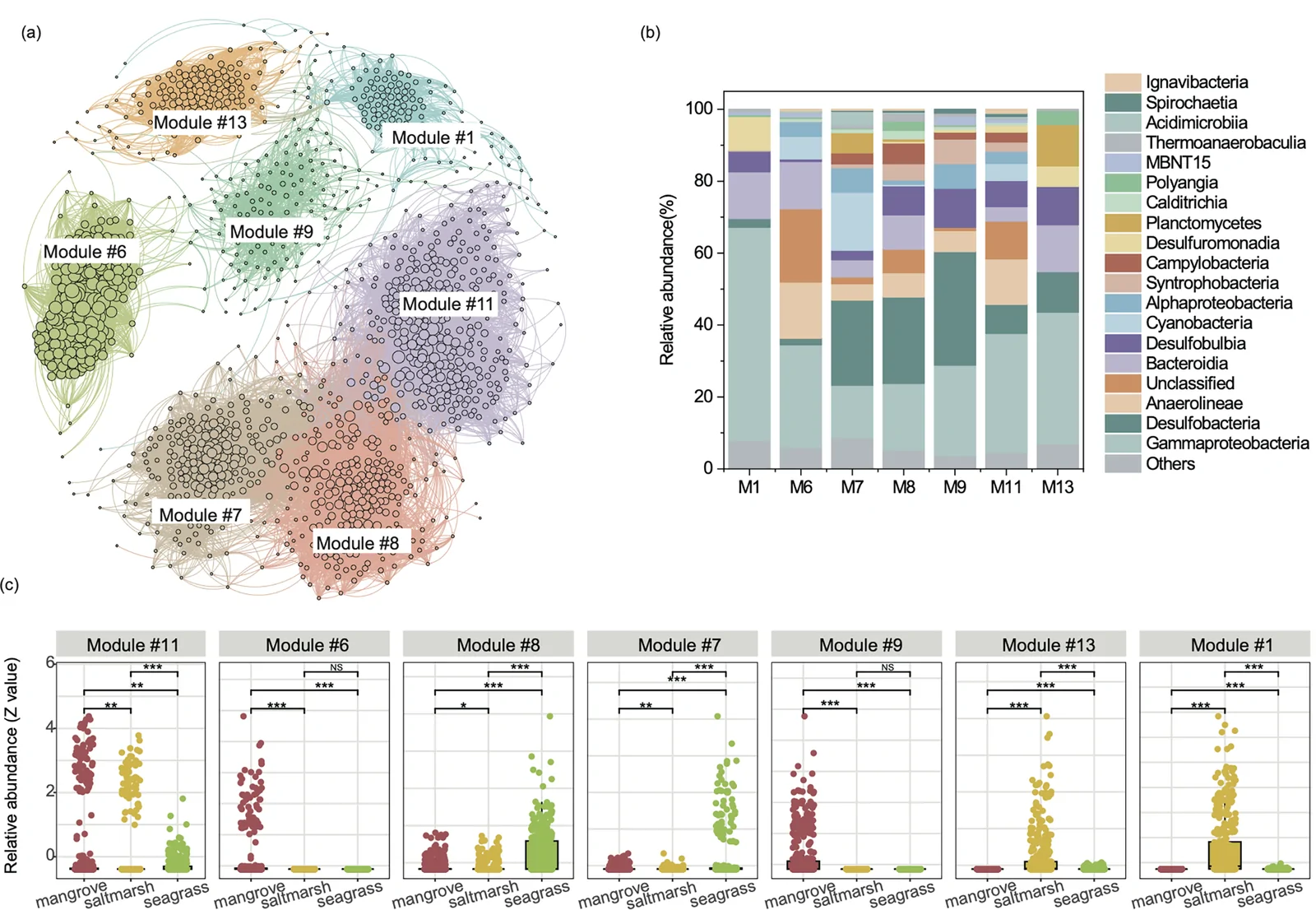
Global ecological networks and module characteristics across three coastal wetlands. (**a**) Co-occurrence network highlighting major modules (clusters of highly interconnected taxa) containing more than 500 nodes. The size of each node is proportional to its degree (the number of significant connections), and node colors represent different modules. (**b**) Microbial community composition at the class level for the dominant ecological modules. (**c**) Comparisons of the relative abundance (Z-score transformed) of each ecological module across mangrove, saltmarsh, and seagrass habitats. Significant differences were determined using the Wilcoxon rank-sum test (*P < 0.05, **P < 0.01, ***P < 0.001; NS, not significant).

Co-occurrence network analysis was employed to identify and characterize the ecological interactions and clustered organization of bacterial taxa within the three coastal wetlands. The constructed network was robust, consisting of 1,521 nodes and 64,463 edges, with a substantial representation of major nodes from the phyla *Gammaproteobacteria*, *Desulfobacteria*, *Bacteroidia,* and *Desulfobulbia* (Fig. 4). The network displayed significant modularity, with seven prominent modules each containing over 300 nodes: Module #11 (17.26%), Module #6 (16.17%), Module #8 (12.18%), Module #7 (11.82%), Module #9 (7.72%), Module #13 (7.47%), and Module #1 (6.22%) (Fig. 4a). These modules encompassed diverse bacterial taxa, with *Gammaproteobacteria*, and *Desulfobacteria* being among the most abundant class groups (Fig. 4b). Notably, the relative abundance of these modules was significantly higher in mangroves compared to saltmarshes and seagrasses (*P* < 0.01, Fig. 4c). Specifically, Modules #11, #6, and #9 showed significantly greater abundance in mangroves. Furthermore, Modules #13 and #1 exhibited the highest bacterial taxaon abundance in saltmarshes among the three coastal wetland types (*P* < 0.01), while Modules #8 and #7 had the highest abundances of bacterial taxa in seagrasses.

In addition, we constructed individual association networks for mangrove, saltmarsh, and seagrass ecosystems. The mangrove network contained five major modules, with module #2 being the largest (34.86% of all nodes) and dominated by *Proteobacteria* (Fig. S4a). Saltmarsh and seagrass networks comprised four and five modules, most of which were primarily dominated by *Acidobacteria* and *Proteobacteria*, respectively (Fig. S4b and c). A module represents a group of highly interconnected ASVs, indicating shared niche preferences. All three networks exhibited typical characteristics of microbial co-occurrence networks (Table 1) and displayed well-defined modular structures with modularity indices exceeding 0.40. Among them, the saltmarsh network was the most highly connected, exhibiting the greatest number of links (9,078), highest network density (0.21), and largest average degree (avgK = 16.13), compared with the mangrove (7,279 links; density = 0.17; avgK = 12.82) and seagrass (7,608 links; density = 0.18; avgK = 13.62) networks. In contrast, the mangrove network showed the highest modularity (0.60) and average geodesic distance (GD = 2.30), whereas saltmarsh and seagrass networks showed lower values (modularity = 0.53 and 0.40; GD = 2.09 and 2.03, respectively).

**TABLE 1:** Topological properties of associated random molecular ecological networks in mangrove, saltmarsh, and seagrass sediments worldwide^*a*^

Coastal wetland type	Network properties of coastal wetland ecosystems							
	Nodes	Links	avgCC	GD	HD	avgK	Density	Modularity
Mangroves	298	7,279	0.20	2.30	0.49	12.82	0.17	0.60
Saltmarshes	293	9,078	0.20	2.09	0.57	16.13	0.21	0.53
Seagrasses	293	7,608	0.19	2.03	0.53	13.62	0.18	0.40

^*a*^ avgCC, average clustering coefficient; GD, average path distance; HD, harmonic geodesic distance; avgK, average degree.

### Habitat-driven differences in potential microbial function across mangroves, saltmarshes, and seagrasses

Functional profiling of microbial communities was conducted using Tax4Fun2 based on 16S rRNA gene sequencing data to predict the potential metabolic capabilities across the three ecosystems. As shown in Fig. S6, we found that the predicted functional profiles of the different wetlands were significantly distinct. Specifically, the predicted functional profiles were predominantly assigned to the categories of metabolism (59.44%–86.28%), environmental information processing (4.44%–19.83%), cellular processes (3.29%–13.29%), genetic information processing (2.19%–8.11%), human diseases (0.90%–2.95%), and organismal systems (0.98%–1.88%). Within the metabolism category at KEGG level 2, carbohydrate metabolism (19.82%–35.44%), amino acid metabolism (16.17%–26.41%), and energy metabolism (6.77%–16.65%) were the most abundant subcategories, exceeding the relative abundances of all other metabolic subfunctions.

As shown in Fig. S7, KEGG-based functional predictions revealed significant differences in predicted functional profiles between mangrove and seagrass ecosystems. Between mangroves and seagrasses, mangrove communities showed higher abundances of taxa associated with predicted carbon fixation pathways and aromatic compound degradation, whereas predicted functional sulfur metabolism was more dominant in seagrass communities (Fig. S7a). Compared with saltmarshes, mangrove microbial communities exhibited a higher predicted functional potential related to nitrogen metabolism, sulfur metabolism, and aromatic compound degradation (Fig. S7b). Seagrass-associated microbial communities showed a higher relative abundance of taxa linked to predicted sulfur metabolism and carbon fixation potential functions compared with saltmarsh communities (Fig. S7c). While saltmarsh communities showed a relatively greater predicted functional potential associated with certain aromatic hydrocarbon metabolism (Fig. S7c), these patterns may indicate that coastal wetland habitats harbor distinct predicted functional profiles, potentially reflecting differences in environmental conditions and biogeochemical processes.

### Microbial communities are more shaped by ecological drift in coastal wetland ecosystems

**Fig 5:**
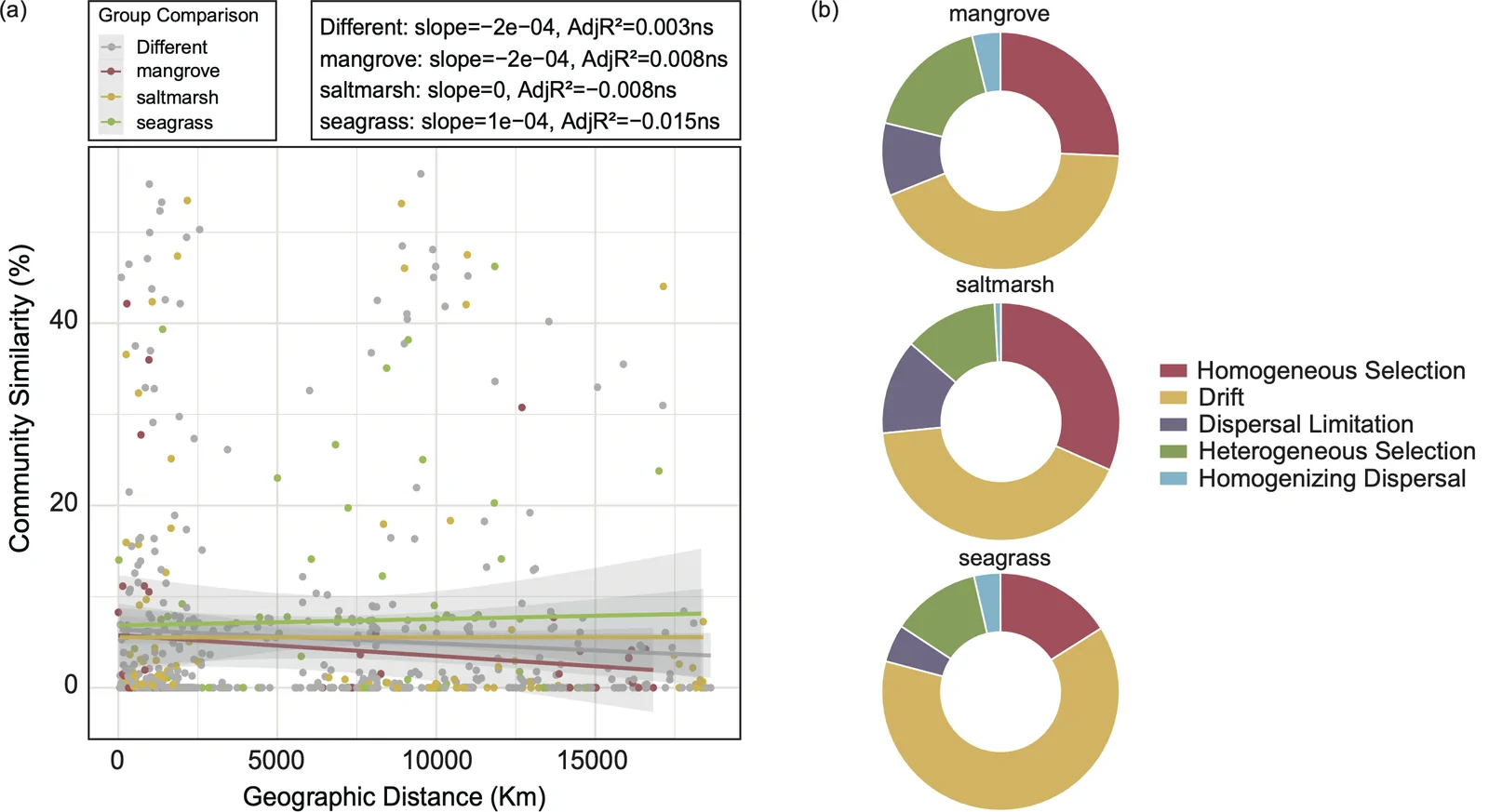
Community assembly and biogeographic patterns across three coastal habitats. (**a**) Distance-decay relationships of microbial communities within and between coastal ecosystems. Linear regressions depict community similarity as a function of geographic distance across mangroves, salt marshes, and seagrasses, as well as between different habitat pairs. Slopes, adjusted R2, and significance levels (ns, not significant) are indicated for each linear fit. (**b**) Relative contributions of ecological processes governing community assembly inferred using a null model framework across the three habitats.

Investigating these microbial mechanisms at a large spatial scale offers comprehensive insights into their universal characteristics and helps elucidate microbial interaction patterns. Our analysis revealed that geographic distance does not significantly influence community similarity across the top 50 ASV groups in mangroves, saltmarshes, or seagrasses (Fig. 5a; *P* > 0.05). All three ecosystems exhibited near-zero regression slopes and non-significant adjusted R² values, indicating that community composition was independent of spatial separation. This finding suggests that large-scale dispersal limitation played a limited role in structuring microbial communities across these coastal ecosystems. Although saltmarshes and mangroves displayed weak negative trends, seagrasses exhibited the weakest spatial signal (Adj. R² = −0.015), highlighting minimal spatial autocorrelation and a nearly absent distance-decay relationship.

Furthermore, the null model was employed to quantify the influence of different ecological processes on microbial community assembly in mangroves, saltmarshes, and seagrasses (Fig. 5b). The analysis identified ecological drift, homogeneous selection, and heterogeneous selection as the primary drivers across these coastal wetlands. As illustrated in Fig. 5b and Table 2, drift was the dominant ecological process across all CWEs. Specifically, drift was the most pronounced assembly process in seagrass communities (63.10%), whereas its contribution was lower but remained substantial in mangroves (43.17%) and saltmarshes (41.80%). Deterministic processes, including homogeneous and heterogeneous selection, also contributed considerably to community assembly in mangroves and saltmarshes. The combined contribution of deterministic selection reached 43.04% in mangroves and 44.42% in saltmarshes, compared with 28.15% in seagrass sediments. Among the deterministic processes, homogeneous selection represented the largest component in both saltmarshes (31.65%), whereas mangroves exhibited the highest contribution of heterogeneous selection (17.34%), compared with saltmarshes (12.77%) and seagrasses (12.12%), suggesting greater environmental heterogeneity within mangrove habitats. The low contribution of dispersal limitation across all three ecosystems explains the absence of significant distance-decay relationships, suggesting that environmental filtering and random demographic events are the principal architects of these microbial landscapes.

**TABLE 2:** Proportion of the assembly processes in different coastal wetland ecosystems

Ecological process	Mangroves	Saltmarsh	Seagrasses
Heterogeneous selection	17.34%	12.77%	12.12%
Homogeneous selection	25.70%	31.65%	16.03%
Dispersal limitation	9.92%	12.90%	5.14%
Homogenizing dispersal	3.87%	0.88%	3.61%
Ecological drift	43.17%	41.80%	63.10%

## DISCUSSION

### Distinct characteristics of bacterial taxa in mangroves, saltmarshes, and seagrasses

Habitat transformation is a key driver of microbial communities, influencing their composition, assembly, and network interactions. While these aspects have been studied across various ecosystems, analyzing global coastal wetlands can reveal overarching ecological principles that extend beyond insights from individual studies. To address this, we leveraged a large-scale global data set of 1,381 samples to compare the diversity, composition, potential functions, and assembly mechanisms of microbial communities across mangroves, saltmarshes, and seagrasses. Our results revealed that *Proteobacteria*, *Actinobacteria*, *Bacteroidetes*, *Firmicutes*, *Acidobacteria*, and *Planctomycetes* were the dominant phyla in CWEs, which is consistent with previous findings (34, 35). A previous study reported a distinct microbial consortium in intertidal wetland sediments, encompassing both heterotrophic and saprophytic groups (e.g., *Actinomycetales*, *Bacteroidetes*, *Firmicutes*, and *Gammaproteobacteria*) and key primary producers (e.g., *Chloroflexi*) (4). This assemblage highlights the coexistence of decomposers and functional specialists, underscoring the complex interactions that sustain biogeochemical processes in intertidal wetland sediments.

In this study, significant variation in microbial composition was observed across the three CWEs (Fig. 2), echoing findings from several studies that have highlighted considerable microbial differences in various coastal wetlands (36–38). For example, the mangroves-associated microbial communities have a distinct profile, with significantly higher abundances of phyla like *Chloroflexi*, *Gemmatimonadota*, and *Latescibacterota* compared to saltmarshes and seagrasses (Fig. 2b), which aligns with earlier reports on mangrove sediment microbiomes (21, 39, 40). Additionally, our analysis identified Rhodothermia and Anaerolineaceae as core taxa, acting as a predominant family and a central hub in the microbiome of mangroves. Their significant influence on community assembly underscores their critical role, especially in mangrove ecosystems (22). In this study, *Anaerolinea* (phylum *Chloroflexi*) was identified as a highly abundant genus and a potential key biogeochemical linker in coastal sediments. Previous research has shown that members of *Anaerolineae* encode and express diverse pathways related to carbon degradation and carbon fixation (41). Furthermore, earlier studies have characterized *Anaerolinea* as versatile anaerobes capable of degrading complex carbohydrates and cellular debris under anoxic conditions (42). Consistent with earlier findings in mangrove sediments (40), *Anaerolinea* not only dominated the microbial community but also acted as a main taxon within species interaction networks. These findings indicate that *Anaerolinea* may function as a central biogeochemical linker that contributes to energy flow and ecosystem stability in mangrove sediments (40, 43).

In saltmarshes, *Proteobacteria* (38%), *Bacteroidota* (15%), and *Cyanobacteria* (2%) were significantly higher than those of mangroves and seagrasses (Fig. 2). *Proteobacteria* is generally considered the largest bacterial phylum in saltmarsh ecosystems (44). Although *Proteobacteria* consistently dominate as the most abundant phylum, their compositional profile exhibits considerable variation in response to environmental conditions (45). For instance, in saltmarsh ecosystems, the microbial community is enriched with *Proteobacteria* (such as *Nitrosomonas*, *Pseudomonas*, and *Geobacter*) and *Nitrospira* (Phylum *Nitrospirota*), which play essential roles in nitrification, denitrification, and organic matter decomposition (46). Meanwhile, *Cyanobacteria* are widely distributed in brackish marshes, where they act as essential primary producers and contribute to nitrogen availability through nitrogen fixation (46, 47). Other phyla, *Bacteroidota* was also the most abundant phylum in saltmarshes that appeared to be directly correlated with salinity (48). These findings highlight how the convergence of diverse metabolic groups sustains nutrient cycling and productivity in coastal wetland sediments.

The microbial community of seagrasses exhibited a distinct composition, with a high abundance of *Desulfobacterota*, *Firmicutes*, *Cyanobacteria*, and *Spirochaetota* when compared to other coastal wetlands. This observation is consistent with previous research showing that these phyla are dominant in root microbiomes (49, 50). The presence of *Spirochaetota* (a sulfur-oxidizing taxon) and *Desulfobacterota* (a sulfate-reducing taxon) on roots confirms the critical importance of sulfur cycling in the eelgrass rhizosphere (51). Our results indicated that the bacterial communities in the sediment surrounding seagrasses differ significantly from those found in mangroves and saltmarsh sediments. The previous study also confirmed that plant-driven changes in the rhizosphere, caused by radial oxygen loss, are responsible for altering the surrounding microbial community composition (52). In summary, the microbial identity of each ecosystem is defined by its dominant groups: mangroves are characterized by anaerobic *Chloroflexi* and *Anaerolineaceae*; saltmarshes by high abundances of *Proteobacteria* and *Bacteroidota*; and seagrasses by sulfur-active *Desulfobacterota* and *Spirochaetota*.

### Core taxa and their potential functions in mangroves, saltmarshes, and seagrasses

Although Tax4fun2 predictions are derived from taxonomic profiles rather than direct functional or metagenomic measurements, they provide a preliminary baseline for understanding putative metabolic capabilities within these sediment bacterial communities (53). We found that the most dominant species in each habitat exhibited a modular co-occurrence pattern. Specifically, these modules were consistently characterized by dominant phylotypes from the classes *Gammaproteobacteria*, *Desulfobacteria*, *Anaerolineae,* and *Bacteroidia* across all habitats (Fig. 4 and Fig. S4), suggesting that these prevalent phylotypes may be specifically adapted to CWE conditions. The formation of tightly connected ecological clusters further implies the presence of potentially coordinated interactions that could contribute to key biogeochemical processes (54–56).

Network topology differed substantially among ecosystem types (Fig. S4, Table 1). The saltmarsh network exhibited the greatest structural complexity, with higher connectivity than the mangrove and seagrass networks. This result suggests a more densely interconnected microbial community and stronger opportunities for metabolic interdependence among taxa (57, 58). However, although high connectivity can enhance theoretical community coordination, it may also theoretically increase the network’s vulnerability (59). Mangrove networks showed the highest modularity but the lowest connectivity, indicating that microbial interactions were more compartmentalized and concentrated within individual modules (60). Meanwhile, the seagrass network exhibited greater overlap among modules, suggesting more extensive cross-module associations throughout the community. This relatively integrated network structure may promote ecosystem-wide connectivity but could also increase sensitivity to disturbances affecting key taxa or interactions (61).

Random forest modeling identified biomarkers across the three CWEs (Fig. 3b). In mangrove ecosystems, the class *Deferrisomatia* was identified as a dominant taxon, which has been reported to possess anaerobic dissimilatory iron-reducing capabilities (62). The potential role of *Desulfurococcales* as a carbon-fixing taxon is hypothesized to contribute to the function of mangroves as key blue carbon sinks (54). The putative biomarker taxa, such as *Anaerolineae*, *Firmicutes*, and *Chloroflexi*, are consistent with those previously reported as dominant taxa in mangrove sediments (40). Furthermore, rare taxa such as *Fibrobacterota* are hypothesized to drive cellulose hydrolysis (20), implying that such taxa may play specialized functional roles in microbiome assembly. In seagrass ecosystems, *Desulfobacterota* and *Actinobacteria* were identified as central phylotypes, which are also considered critical for maintaining network stability (Fig. S4). The enrichment of *Campylobacterota* and *Desulfocapsaceae* was also observed (Fig. 3b). While *Desulfocapsaceae* are putative sulfate-reducing bacteria (63), *Campylobacterota* are likely involved in sulfide oxidation. These taxa may play a vital role in coupling sulfur cycling with carbon fixation and mitigating sulfide toxicity in marine plant rhizospheres (55, 64). Overall, the inferred functional profiles also suggest distinct metabolism across CWEs: mangroves appear driven by putative carbon fixation and organic matter degradation, saltmarshes by complex metabolic cooperation within dense networks, and seagrasses by specialized sulfur-cycling consortia that may contribute to localized detoxification.

### Alterations in microbial biogeographic patterns and community assembly in response to the different coastal wetland habitats

CWEs host vast communities of microorganisms that drive fundamental ecological processes. These microorganisms constantly interact with one another, and these potential ecological interactions are a core driver of how bacterial communities assemble. This complex network of interactions gives rise to the highly dynamic, self-organizing nature of these microbial communities. To understand how microbial communities evolve their functions in response to environmental changes, it is essential to examine their ecological assembly processes (65). Concurrently, microbial communities in both mangroves and seagrasses exhibited weak DDRs (Fig. 5a), indicating less pronounced spatial variability in these ecosystems. Additionally, analysis of microbial diversity across climate zones revealed a significant increase in saltmarshes from temperate to tropical regions (Fig. S8). In contrast, mangroves and seagrass ecosystems exhibited no obvious diversity differences across the climatic regions. These findings align with the established understanding that microbial community composition is shaped by the interplay of historical legacy and contemporary environmental heterogeneity (22, 33).

Furthermore, we revealed ecological drift exhibited the highest relative influence (63.1%) in structuring seagrass microbial assemblages (Fig. 5b). This high contribution of stochasticity is consistent with prior findings that identified drift as the dominant assembly force in seagrass ecosystems (15, 66, 67). However, this result stands in contrast to reports suggesting that seagrass bacterial communities in the rhizosphere are governed predominantly by deterministic processes (20). This discrepancy can be reconciled by considering the distinction between bulk sediments and rhizosphere effects. Seagrasses may actively recruit specific functional groups through root exudates to create a highly selected rhizosphere microbiome. Consequently, the deterministic signal of plant recruitment is likely localized to the immediate root-soil interface, whereas the broader sediment remains dominated by stochastic drift (67).

For mangroves, the dominant ecological processes shaping microbial community assembly were distinctly different from those in seagrasses, with homogeneous and heterogeneous selection playing a significant role (Fig. 5b). This suggests that a strong habitat filtration effect is imposed on the microbial community of mangrove sediments. Our findings are corroborated by (68), who reported that deterministic processes (e.g., variable selection) were a significant influence on the assembly of mangrove *Myxococcota* communities. This pattern can be contextualized by the unique ecological niche of mangroves, which exist at the interface of terrestrial and marine systems. The continuous influence of ocean currents may lead to a relatively homogenized microbial community throughout the sediments, potentially due to reduced dispersal limitation (69).

Interestingly, microbial community assembly in saltmarshes differed significantly from that in seagrasses, with homogeneous selection being the dominant process (Fig. 5b), indicating that microbial taxa best adapted to the local environment are selectively favored. This suggests that species best adapted to the saltmarsh environment are selectively favored, leading to community homogenization (70). Furthermore, our results are also corroborated by (56), who reported that deterministic processes (specifically, homogeneous selection) were the principal drivers of bacterial community assembly in saltmarshes. They explained that the bacterial community becomes more homogeneous following the invasion of smooth cordgrass (71, 72). These findings reinforce the recognized strong influence of plant community composition on bacterial community structure in saltmarsh ecosystems (48, 54). In summary, the assembly mechanisms reveal a gradient of environmental influence. The seagrass communities are primarily structured by stochastic ecological drift, whereas mangroves and saltmarshes are governed by deterministic selection and habitat filtration.

### Limitation

Although this study generated several important insights based on 1,381 samples collected from mangrove, saltmarsh, and seagrass ecosystems, several limitations should be acknowledged. First, the analyzed data sets were collected from multiple regions worldwide, but the available data were unevenly distributed geographically, with a higher proportion originating from Asia. Consequently, regional variation in microbial community composition and assembly processes may not be fully represented. Therefore, caution should be exercised when extrapolating these findings to a global scale. Second, inherent heterogeneity stemming from study identities and sampling methodologies represents an inescapable challenge in large-scale meta-analyses. In this study, although all included samples were sediment-associated and rhizosphere/non-rhizosphere sediments were classified following consistent conceptual definitions across original studies, minor variations in field sampling protocols may have introduced a degree of classification uncertainty. Furthermore, while ComBat-seq batch correction effectively mitigated the primary technical bias introduced by different amplified hypervariable regions (V3–V4 vs. V4), and all raw sequences were harmonized through a standardized QIIME 2 pipeline, potential residual variance from individual study identities and methodological differences could not be completely eliminated. Third, the present analysis was focused on three major coastal blue carbon ecosystems (mangroves, saltmarshes, and seagrasses). Microbial communities inhabiting ecosystems, such as mudflats and coral reefs, may exhibit distinct ecological characteristics that were not captured in this study. Therefore, future studies incorporating a broader diversity of coastal habitats, more geographically balanced data sets, and standardized sampling protocols will be essential for evaluating the generality of the patterns observed here and for developing a more comprehensive understanding of microbial community dynamics and ecosystem functioning across coastal environments.

### Conclusion

Based on a global comparative analysis, this study reveals that mangrove, saltmarsh, and seagrass ecosystems host distinct microbial communities characterized by unique diversity, taxonomic composition, and functional potentials. *Gammaproteobacteria*, *Desulfobacteria*, *Bacteroidia*, and *Desulfobulbia* emerged as dominant taxa across all three CWEs. Mangroves supported the highest microbial diversity, with Rhodothermia, Anaerolineaceae, and SBR1031 identified as indicator taxa. KEGG-based functional predictions suggested that microbial communities across CWEs shared core metabolic features while exhibiting putative habitat-specific functional differences. Ecological drift dominated community assembly in seagrasses, whereas deterministic selection contributed substantially in saltmarshes and mangroves, indicating that microbial community assembly is habitat-dependent. Collectively, these findings advance our understanding of coastal sediment microbiomes and provide inferred functional insights into their potential contributions to biogeochemical cycling in coastal wetlands.

## Data Availability

The newly generated 16S rRNA gene sequencing data sets from Jiangsu coastal saltmarsh sediments have been deposited in the NCBI Sequence Read Archive (SRA) under accession numbers SRR40345552–SRR40345687 and SRR40350353–SRR40350362. Accession numbers for all public microbial data sets analyzed in this study are listed in Data Set S1. All supporting data and materials are available within the article and its supplemental material or from the corresponding author upon reasonable request.

## SUPPLEMENTAL MATERIAL

Data Set S1 aem.01159-26-s0001.xlsx Metadata for 1,235 collected sequencing samples, including NCBI accession numbers and associated source information.

Supplemental material aem.01159-26-s0002.docx Tables S1 to S3; Fig. S1 to S8.

ASM does not own the copyrights to Supplemental Material that may be linked to, or accessed through, an article. The authors have granted ASM a non-exclusive, world-wide license to publish the Supplemental Material files. Please contact the corresponding author directly for reuse.
