# Supplementary material for: Comparative analysis reveals distinct microbial community profiles and assembly mechanisms in sediments of coastal wetland ecosystems
Source: Appl Environ Microbiol. 2026 Sep 23;92(10):e01159-26. doi: 10.1128/aem.01159-26 (PMC13689362; doi:10.1128/aem.01159-26)
Supplement: Supplementary file 1 [file aem.01159-26-s0002.docx]

**Supplementary Materials**

for

**Comparative analysis reveals distinct microbial community profiles and assembly mechanisms in sediments of coastal wetland ecosystems**

Linqiong Wang ^a, b^, Zhen Chen ^a, b^, Xinle Li ^a, b^, Zhe Zhao ^a, b, *^

^a^ College of Oceanography, Hohai University, Nanjing, China;

^b^ Jiangsu Province Engineering Research Center for Marine Bio-resources Sustainable Utilization, Hohai University, Nanjing, China.

Correspondence to:

Prof. Zhe Zhao

College of Oceanography, Hohai University, Xikang Road #1, Nanjing210024, China.

Email: zhezhao@hhu.edu.cn

**List of supplementary information:**

**Table S1** Summary of datasets included in the meta-analysis and their sampling periods.

**Table S2** Quantification of sequencing-region-associated variation before and after batch correction based on PERMANOVA analysis.

**Table S3** PERMANOVA results based on weighted UniFrac distances.

**Fig. S1** Seasonal (a) and temporal (b) distribution of sampling projects across habitat types.

**Fig. S2** Flowchart of the literature search and study selection process for the meta-analysis.

**Fig. S3** Principal Coordinates Analysis (PCoA) of microbial community composition before and after batch correction. PCoA plots based on BraycCurtis dissimilarities calculated from family-level taxonomic profiles. Left panels show the original data before correction, and right panels show the data after ComBat-Seq correction. In the upper panels, samples are colored by primer region (V3–V4 and V4). In the lower panels, samples are colored by habitat type (mangrove, saltmarsh, and seagrass), with dashed ellipses representing habitat groupings.

**Fig. S4** Network associations of bacterial ASVs in the three coastal wetlands. The networks represent random matrix theory-based correlation models derived from all samples for each dataset, where nodes represent ASVs, and links between the nodes represent significant correlations. The node size is proportional to its degree, and the modules indicate the groups of taxa highly co-occurring with each other.

**Fig. S5** The cross-validation error as a function of the number of input classes used to classify against group.

**Fig. S6** Functional annotation and pathway distribution based on the KEGG database. (a) KEGG Level 1 functional categories. (b) KEGG Level 2 metabolism pathways.

**Fig. S7** The variations of functional potentials of microbiomes in the mangrove forests, saltmarshes and seagrass beds. (a) mangrove forests vs seagrass beds, (b) mangrove forests vs Saltmarshes, (c) seagrass beds vs saltmarsh.

**Fig. S8** Similarity analysis of microbial communities across different temperature bands in three coastal wetlands (Wilcoxon test, ns: not significant, *p* > 0.05; **: highly significant, *p* < 0.01)

**Table S1.** Summary of datasets included in the meta-analysis and their sampling periods.

| Project | Habitat | Time |
| --- | --- | --- |
| PRJNA282077 | seagrass | 2013(6\9) |
| PRJNA433971 | saltmarsh | 2013(7\10) |
| PRJNA283936 | mangrove | 2013(8\10\11) |
| PRJNA387284 | saltmarsh | 2014(9)-2015(3\8\10) |
| PRJNA497291 | seagrass | 2015(5\6)-2016(6\8) |
| PRJNA393594 | mangrove | 2016(1) |
| PRJNA574590 | seagrass | 2016(2\4) |
| PRJNA813530 | mangrove | 2016(5\8\10)-2017(2\3\8) |
| PRJDB8775 | seagrass | 2016(7) |
| PRJNA511001 | seagrass | 2017(11) |
| PRJNA765018 | saltmarsh | 2017(5\7\10)-2018(1) |
| PRJEB48899 | seagrass | 2017(7)-2018(10) |
| PRJNA475455 | mangrove | 2017(9\12) |
| PRJNA649070 | seagrass | 2018(10) |
| PRJNA666636 | saltmarsh | 2018(7)-2019(7) |
| PRJNA854764 | saltmarsh | 2018(8)-2019(10) |
| PRJNA1039151 | mangrove | 2019 |
| PRJNA733060 | saltmarsh | 2019(1) |
| PRJNA612579 | mangrove | 2019(4) |
| PRJNA545746 | seagrass | 2019(6) |
| PRJNA613355 | seagrass | 2019(6) |
| PRJNA755846 | saltmarsh | 2019(6\7\8) |
| PRJNA646607 | seagrass | 2019(9) |
| PRJNA985394 | seagrass | 2021(6) |
| PRJNA961635 | mangrove | 2022(8) |
| PRJNA1150947 | saltmarsh | 2022-2023 |

**Table S2** Quantification of sequencing-region-associated variation before and after batch correction based on PERMANOVA analysis.

|  | Df | Sum Of Squares | R^2^ | Pseudo-F | P-value |
| --- | --- | --- | --- | --- | --- |
| Primer (before) | 1 | 38.20 | 0.08326 | 136.007 | 0.001 *** |
| Primer (after) | 1 | 11.24 | 0.02495 | 37.316 | 0.001 *** |
| Group (before) | 2 | 22.36 | 0.04873 | 39.803 | 0.001 *** |
| Group (after) | 2 | 21.25 | 0.04719 | 35.287 | 0.001 *** |

Abbreviations: Df, degrees of freedom; R², proportion of variance explained; Pseudo-F, PERMANOVA test statistic. P-values were obtained from permutation tests (999 permutations), *** indicates p < 0.001.

**Table S3** PERMANOVA results based on weighted UniFrac distances.

| **factors** | DF | Sum of squares | R² | F | p-value |
| --- | --- | --- | --- | --- | --- |
| **Group** | 2 | 3.1931 | 0.118 | 82.032 | 0.001 *** |
| **Residual** | 1227 | 23.8805 | 0.882 |  |  |
| **Total** | 1229 | 27.0736 | 1 |  |  |

**Fig. S1** Seasonal (a) and temporal (b) distribution of sampling projects across habitat types.

**Fig. S2** Flowchart of the literature search and study selection process for the meta-analysis.

**Fig. S3** Principal Coordinates Analysis (PCoA) of microbial community composition before and after batch correction. PCoA plots based on BraycCurtis dissimilarities calculated from family-level taxonomic profiles. Left panels show the original data before correction, and right panels show the data after ComBat-Seq correction. In the upper panels, samples are colored by primer region (V3–V4 and V4). In the lower panels, samples are colored by habitat type (mangrove, saltmarsh, and seagrass), with dashed ellipses representing habitat groupings.

**Fig. S4** Network associations of bacterial ASVs in the three coastal wetlands. The networks represent random matrix theory-based correlation models derived from all samples for each dataset, where nodes represent ASVs, and links between the nodes represent significant correlations. The node size is proportional to its degree, and the modules indicate the groups of taxa highly co-occurring with each other.

**Fig. S5** The cross-validation error as a function of the number of input classes used to classify against group.

**Fig. S6** Functional annotation and pathway distribution based on the KEGG database. (a) KEGG Level 1 functional categories. (b) KEGG Level 2 metabolism pathways.

**Fig. S7** The variations of functional potentials of microbiomes in the mangrove forests, saltmarshes and seagrass beds. (a) mangrove forests (red) vs seagrass beds (green), (b) mangrove forests vs saltmarshes (yellow), (c) seagrass beds vs saltmarsh.

**Fig. S8** Similarity analysis of microbial communities across different temperature bands in three coastal wetlands (Wilcoxon test, ns: not significant, *p* > 0.05; **: highly significant, *p* < 0.01)
